# The Interpretation of Diffuse Reflectance Spectra

**DOI:** 10.6028/jres.080A.056

**Published:** 1976-08-01

**Authors:** Harry G. Hecht

**Affiliations:** Department of Chemistry, South Dakota State University, Brookings, South Dakota 57006

**Keywords:** Absolute absorptivities, continuum models, diffuse reflectance, radiative transfer, reflectance spectra, scattering coefficients, statistical models

## Abstract

Numerous treatments of the diffuse reflecting properties of scattering media have been described. Many theories give an adequate account of the reflectance for a specific set of conditions for which the model was constructed and the solution tested experimentally. Only those models which are considered to be fairly general are considered here.

It is convenient to divide the theories into those based upon continuum models and those based upon statistical models. The continuum models typically describe the scattering and absorbing properties of a given medium in terms of two phenomenological constants. These models may all be regarded as varying levels of approximate solution to the general equation of radiative transfer. This provides a convenient basis for comparison of the various theories.

The statistical models are based upon a summation of transmittances and reflectances from individual layers or particles. Thus, some assumptions must be made about the nature of the fundamental units, and the validity of the ultimate result will depend upon how closely these assumptions correspond with reality. Only the statistical models lead to expressions from which absolute absorptivities and scattering coefficients can be calculated and related to the actual particle characteristics.

The relationship between the various models will be discussed and the features which typify the absorptivity and scattering coefficient according to each will be compared and related to the available experimental data. This leads to a consideration of the characteristics of appropriate model systems and standards.

## I. Introduction

It is now recognized that diffuse reflectance spectroscopy is a very useful companion technique to transmission spectroscopy. Not only can it provide absorption data in some cases where transmission measurements fail, but for many industrial and research applications, it may in fact be the preferred technique.

Our discussion will be concerned with the behavior of radiation within a scattering medium. For simplicity we assume the scattering centers, which may also absorb radiation, are imbedded in a medium which neither scatters nor absorbs. The medium may usually be taken as air, although there are many other cases of interest in which the refractive index of the medium is much greater than unity. We will not deal specifically with these cases in the present review, nor with those processes which alter the frequency of the radiation, such as luminescence and fluorescence.

Scattering takes place under a wide variety of conditions. One may be concerned with the glowing photosphere of the sun, which is surrounded by a cloud of electrons that reradiate the direct sunlight incident on them. Sunlight is also reradiated by cosmic dust, which accounts for the outer part of the corona.

In a more down-to-earth situation one may be dealing in the laboratory with the spectroscopy of a powder, paper, opal glass, photographic emulsion, etc. As so often happens, concepts developed in one area of science are slow to find their way into another. We will attempt to show that there is a close relationship between the astrophysical solutions, which are based largely on radiative transfer theory, and the various models which are more familiar to the spectroscopist.

Since we will not be following a historical development, it may be useful to point out some relationships between the early studies. The first attempt to account for transmission and reflection of a layered material was carried out by Stokes in about 1860 [1],^1^ and led to some very useful relationships which have also been derived by other workers (vide infra). Lord Rayleigh [2] and Mie [3] developed the theory of single scatter to a high degree, but Schuster [4] was the first to consider multiple scatter. He was concerned with the cloudy atmospheres of stars, and developed a plane-parallel plate model in which the radiation field was divided into forward and backward components. This same model was used much later by Kubelka and Munk [5], whose names are usually attached go it by spectroscopists. Schwarzs-child [6] showed that the radiation field should be characterized by a complete angular distribution, and if one integrates over the forward and backward hemispheres, the Schuster model is obtained as a first approximation. A further generalization of the Schwarzschild formulation leads to an integro-differential equation known as the equation of radiative transfer, which is very general in concept, but can be solved exactly in only a few cases.

The radiative transfer theory and various models stemming from it are referred to as Continuum Models. They have in common the characterization of the scattering and absorbing properties of the medium through phenomenological constants, usually two in number. These theories will be considered in section II. A completely satisfactory theory must of course relate the measurable quantities to fundamental properties of the scattering material, such as particle size, refractive index, and absorptivity. This is the goal of the statistical theories. Many have been proposed; those which show the most promise are discussed in section III. In section IV we discuss some of the strengths and weaknesses of the various models and some relationships between them. Section V considers the meaning of the absorption and scattering coefficients often used to interpret reflectance data, as well as the characteristics required of appropriate model systems.

We will limit our discussion to ideal, homogeneous dispersions. There have been interesting developments in the theory of nonhomogeneous media, mixtures, luminescing materials, diffusing media in which photochemical reactions are taking place, and reflectance for materials dispersed in a highly refracting matrix, but these topics are considered to be outside the scope of the present review. Many of these topics are covered in the books which have been written on the subject [7,8,9]. Further information has been presented at various symposia which have been held, and the proceedings of at least two of them are available [10,11]. These sources, together with various reviews devoted to applications in particular fields, have made workers aware of the power of the technique. Thus we will confine ourselves to certain theoretical aspects.

The present paper may be regarded as an update to a former review of the subject by the author [12]. Most of the theoretical work of the intervening years has been in the development of more refined statistical models, and in showing the relationship between the various theories. At present there is some reason to feel optimistic that out of a morass of apparently divergent and unrelated theory, some order is beginning to emerge.

## II. Continuum Theory

As is often the case with applications of physical theory, the real media with which diffuse reflectance spectroscopy is concerned are intermediate between two extreme cases, each of which is well understood. The one limiting case involves the propagation of radiation through quasi-homogeneous matter, where the radiation field is characterized by smoothly and slowly varying functions of the coordinates. The other extreme involves the emission, absorption, and scattering of radiation by single particles in a homogeneous medium. The local transformation of the radiation field at the boundaries of quasihomogeneous media is a special case which has likewise been characterized for a long time, and leads to such well-known phenomena as reflection, refraction, etc.

In dense scattering media, it is important to consider the manner in which the radiation fields from the various scattering centers interact with one another. Rozenberg [13, 14, 15] has pointed out that the interaction can be treated as a sum of two parts, one of which is coherent and the other incoherent. The coherent part is largely due to nearest neighbor interactions and gives rise to dispersion effects; i.e., is involved with changes of refractive index. The incoherent part is that with which we are primarily concerned in the present discussion. It involves multiple scattering as a sum of interactions throughout the entire medium. In most treatments the multiple scattering, because of its complexity, is separated rather arbitrarily from dispersive effects. Such a separation reduces the problem to one involving geometrical optics, and allows one to write a radiative transfer equation in which the absorbing and scattering properties of the medium are treated as phenomenological constants (spoken of as "external parameters” by Stepanov[16]).

The concept that each volume element is irradiated by scattering from every other volume element of the medium (The Principle of Self-Illumination), is a basic concept of radiative transfer theory which clearly pervades the writings of such early workers as Schuster [4, 17], King [18], and Schwarzschild [6]. The equation of radiative transfer can in fact be regarded as a simple statement of the law of conservation of energy. The change in intensity of a beam along its direction of propagation, *dI*, is equal to the radiation which is lost through absorption and scattering –*κρIdx* plus that which is scattered in this direction from all other directions, *jρdx*:

dI=−κρIdx+jρdx (1)

Here *ρ* is the density, *κ* is the attenuation coefficient, *j* is the scattering function, and *dx* is the element of path length. The scattering function can be written as

$$j(\theta ,\phi )=\frac{\sigma }{4\pi }\int_{0}^{\pi }\int_{0}^{2\pi }p(\theta ,\phi ;\theta^{\prime },\phi^{\prime })I(\theta^{\prime },\phi^{\prime })I(\theta^{\prime },\phi^{\prime })\sin\theta^{\prime }d\theta^{\prime }d\phi^{\prime }$$ (2)

where *σ* is the scattering coefficient and *p*(*θ,ϕ*; *θ′,ϕ*′) is the phase function which defines the probability that radiation which travels initially in the direction (*θ′,ϕ*′) is scattered into the direction (*θ,ϕ*). Equation (1) is usually solved in terms of the optical thickness

$$\tau =\int_{x_{1}}^{x_{2}}\kappa \rho dx$$ (3)

Defining *θ* as the angle with respect to the inward surface normal gives

$$\mu \frac{dI(\tau ,\mu ,\phi )}{d\tau }=I(\tau ,\mu ,\phi )-J(\tau ,\mu ,\phi )$$ (4)

where *µ* = cos *θ* and *J=j*/*σ* is the source function.

The equation of radiative transfer can be generalized to include dispersion effects as well as scattering [14, 15]. In terms of the components of the Stokes vector, I,

$$\begin{array}{l} \frac{dI_{i}(\theta ,\phi )}{dx}=\sum_{j=1}^{4}\{-\kappa_{ij}I_{j}(\theta ,\phi )\\ +\frac{\sigma }{4\pi }\int_{0}^{\pi }\int_{0}^{2\pi }p(\theta ,\phi ;\theta^{\prime },\phi^{\prime })I_{j}(\theta^{\prime },\phi^{\prime })\sin\theta^{\prime }d\theta^{\prime }d\phi^{\prime }\}\\ +I_{i}^{\prime }(\theta ,\phi ). \end{array}$$ (5)

The term $I_{i}^{\prime }\left(\theta ,\phi \right)$ accounts for possible self-radiation of the volume element, which is of importance in the infrared spectral region or in luminescing media. As mentioned above, this term will be suppressed in the present discussion.

### A. The Layer Model

A model in which the scattering medium is approximated by plane-parallel layers has been used by numerous workers as a basis for reflectance theory [7, 8]. We consider a pair of adjacent layers, with *R_i_*, *R_j_* and *T_i_*, *T_j_* as the reflectances and transmittances for radiation incident in one direction, and *R_I_, R_J_* and *T_I_*, *T_j_* as the reflectances and transmittances for radiation incident in the other direction.

**Figure 1:**
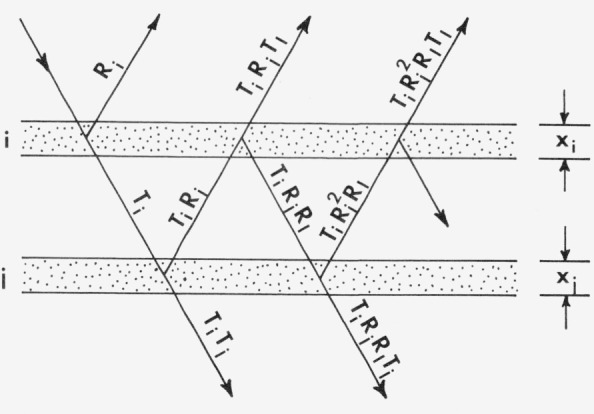
Reflectance and transmittance of a pair of inhomogeneous layers (Kubelka [19]).

If the incident beam is of unit intensity, then the portion *R_i_* is reflected and the portion *T_i_* transmitted by the first layer. At the second layer the portion *T_i_R_j_* is reflected and *T_i_T_j_* is transmitted. The beam *T_i_R_j_* strikes the underside of layer *i* where *T_i_R_j_T_I_* is transmitted, while *T_i_R_j_R_I_* is reflected. Continuing this process indefinitely (see fig. 1) we find that the transmittance and reflectance of the combined layers are given by

$$\begin{array}{l} T_{i+j}=T_{i}T_{j}(1+R_{I}R_{j}+R_{{\;}_{I}}^{2}R_{j}^{2}+\ldots )\\ R_{i+j}=R_{i}+T_{i}T_{I}R_{j}(1+R_{I}R_{j}+R_{I}^{2}R_{j}^{2}+\ldots ) \end{array}$$

Summing the geometric series gives

$$T_{i+j}=\frac{T_{i}T_{j}}{1-R_{I}R_{j}}$$ (6)

$$R_{i+j}=R_{i}+\frac{T_{i}T_{I}R_{j}}{1-R_{I}R_{j}}$$ (7)

We generally deal with the case where *T_i_=T_I_* and *R_i_=R_I_*; i.e., where the reflectance and transmittance of a layer do not depend on the direction of incidence. This is quite a general condition which applies even to the case of nonhomogeneous sheets [19]. With these substitutions eqs (6) and (7) reduce to

$$T_{i+j}=\frac{T_{i}T_{j}}{1-R_{i}R_{j}}$$ (8)

$$R_{i+j}=R_{i}+\frac{T_{i}^{2}R_{j}}{1-R_{i}R_{j}}.$$ (9)

We now make the assumption that the layers are homogeneous and thick compared with individual particle dimensions so that no intrinsic inhomogeneities are evident. We can then write that the reflectance of a given layer is proportional to its thickness [20],

$$R(dx)=K_{1}dx.$$ (10)

Similarly we write

$$T(dx)=1-K_{2}dx.$$ (11)

Here *K*_1_ and *K*_2_ are phenomenological constants which characterize the medium. We assume that *K*_2_*≥K*_1_ where the inequality applies to absorbing media and the equality to nonabsorbing media. We substitute *R*(*x*) and *T*(*x*) for *R_i_* and *T_i_* in eqs (8) and (9), with *R*(*dx*) and *T*(*dx*) from eqs (10) and (11) being substituted for *R_j_* and *T_j_.* Writing *R_i+j_* and *T_i+j_* as *R*(*x + dx*) and *T*(*x+dx*) allows eqs (8) and (9) to be expanded in series to give

$$dR=K_{1}T^{2}dx$$

and

$$dT=-T(K_{2}-K_{1}R)dx.$$

Integrating this set of coupled differential equations subject to the boundary conditions *R*(0)=0, *T*(0) =1 gives

$$R=R_{\infty }\frac{1-e^{-2}Lx}{1-R_{\infty }^{2}e^{-2Lx}}$$ (12)

$$T=(1-R_{\infty }^{2})\frac{e^{-Lx}}{1-R_{\infty }{\;}^{2}e^{-2Lx}}.$$ (13)

*R*_∞_ is the reflectance of an infinitely thick layer, and it is related to *K*_1_ and *K*_2_ through

$$R_{\infty }=\frac{K_{2}-L}{K_{1}}=\frac{K_{2}-\sqrt{K_{2}^{2}-K_{1}^{2}}}{K_{1}}$$ (14)

while *L* is given by

$$L=\sqrt{K_{2}^{2}-K_{1}^{2}}$$ (15)

Equations (12) and (13) were derived long ago by Stokes [1] for plane-parallel plates, with similar subsequent derivations by Schuster [4, 17] and Gurevič [20] for light-scattering layers. These are now known to be a special case of the more general Kubelka-Munk theory [21], which we will now consider.

### B. The Kubelka-Munk Theory

**Figure 2:**
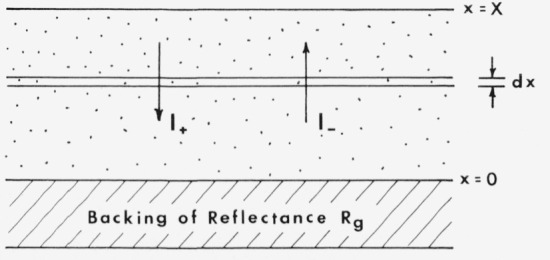
Model for the Kubelka-Munk analysis of reflectance and transmittance of a scattering medium [5].

The Kubelka-Munk theory [5] is based upon a model in which the radiation field is approximated by two fluxes, the one, *I*_+_, traveling from the illuminated sample surface, and the other, *I*_−_ traveling toward the illuminated surface (see fig. 2). As radiation travels from the surface, its intensity is decreased by scattering and absorption processes, both assumed to be proportional to the thickness of the medium traversed. This is partially offset by scattering from the other beam, so we have

$$dI_{+}=-(S+K)I_{+}dx+SI_{-}dx.$$ (16)

The component travelling toward the illuminated surface is similarly described:

$$dI_{-}=(S+K)I_{-}dx-SI_{+}dx.$$ (17)

The constants which we have introduced here are once again phenomenological constants which describe scattering (*S*) and absorption (*K*) within the medium. If we make the following definition,

$$a\equiv \frac{S+K}{S}$$ (18)

we can write

$$\begin{array}{l} \frac{-dI_{+}}{Sdx}=-aI_{+}+I_{-}\\ \frac{dI_{-}}{Sdx}=-aI_{-}+I_{+} \end{array}$$

which can be combined into a single differential equation,

$$\frac{dR}{Sdx}=R^{2}-2aR+1$$ (19)

where *R = I_−_/I_+_.* Equation (19) can be easily integrated over the entire thickness *x* of the scattering medium to obtain [5]

$$R=\frac{\left(R_{g}-R_{\infty })/R_{\infty }-R_{\infty }(R_{g}-1/R_{\infty })\exp[SX(1/R_{\infty }-R_{\infty })\right]}{R_{g}-R_{\infty }(R_{g}-1/R_{\infty })\exp[SX(1/R_{\infty }-R_{\infty })]}$$ (20)

*R_g_* is the reflectance of the background (see fig. 2) and *R*_∞_ is once again the reflectance of a layer which is so thick that further increase in thickness does not alter the reflectance. If eq (19) is integrated over the limits *x*=0 to *x*=∞, a simple formula results [5]

$$R_{\infty }=\underset{x\to \infty }{\lim}R=a-{\left(a^{2}-1\right)}^{1/2}\equiv a-b$$ (21)

Using eq (18) this can be rearranged to give the well-known Kubelka-Munk function *F*(*R_∞_*)

$$F(R_{\infty })\equiv \frac{{\left(1-R_{\infty }\right)}^{2}}{2R_{\infty }}=\frac{K}{S}.$$ (22)

We note in passing that this result follows directly from eq (12) in the limit *x*→∞[23], which once again shows the close relationship of the Gurevič and Kubelka-Munk models.

### C. Rozenberg Solutions

For a homogeneous semi-infinite medium, a good approximate treatment of the reflectance for strong absorbers has been given by Rozenberg [13–15]. The solution involves summing various successive contr butions to the reflecting power by scattering of different degrees of multiplicity, and is based upon concepts developed by Kuznetsov for problems of visibility [23]. To the nth degree of approximation, the reflected intensity in an isotropic medium is given by

$$I_{i}^{\text{ref}}(\theta ,\phi )=I_{1}{\;}^{0}\sum_{t=1}^{n}\frac{a_{it}(\theta ,\phi )}{{\left(1+\beta \right)}^{t}}.$$ (23)

In this case *θ* and *ϕ* define the direction of observation, *I*_1_^0^ is the incident beam intensity, $\beta =\frac{\alpha }{\sigma }$ is the ratio of the absorption and scattering coefficients, and the index *i* runs over the four components of the radiation field (see eq (5)). The *a_it_* coefficients in eq (23) are given by

$$a_{it}=\sum_{j=1}^{4}a_{ijt}(\theta ,\phi )C_{j}^{0}(\theta_{0},\phi_{0})$$

where the $C_{j}^{0}\left(\theta_{0},\phi_{0}\right)\equiv I_{j}^{0}/I_{1}^{0}$ define the polarization of the incident beam, and the *a_ijt_* (*θ*, *ϕ*) are coefficients which depend only on the angles of incidence (*θ*_0_, *ϕ*_0_) and observation (*θ*, *ϕ*), and on the form of the scattering indicatrix.

The reflectance of the medium is given by

$$C_{i}R=\sum_{t=1}^{\infty }\frac{a_{it}}{{\left(1+\beta \right)}^{t}}$$ (24)

where *C_i_=I_i_*/*I*_1_ is the polarization of the reflected radiation. It will be observed that as *β* increases, scattering of higher multiplicities becomes less important. Ambartsumian [24] has shown that the mean multiplicity of scatter in the case of reflection from a semi-infinite turbid medium is $\sqrt{1+1/\beta }$. Thus when *β* ≥ 1/3, a fairly accurate solution is obtained by inclusion of terms up to second or third degree. It is further assumed that the scatter is independent of *β*, which should be a good approximation for mixtures of polydispersive media with different *α* and *σ*; i.e., as with the addition of dye to a suspension. The resulting equation is

$$\frac{R_{0}}{R}\approx \frac{{\left(1+\beta \right)}^{2}}{1+\frac{\beta }{Q}}$$ (25)

where *R*_0_ is the reflectance of the medium itself (when *β*=*β*_0_), and

$$Q=1+\sum_{t=1}^{\infty }\frac{a_{1,t+1}}{a_{11}{\left(1+\beta_{0}\right)}^{t}}.$$ (26)

Thus *Q* is a quantity which defines the relative contribution of higher multiplicities of scattering when *β*=*β*_0_. Both it and *R*_0_ are constants which are independent of the nature and concentration of the colorant.

Equation (25) may be regarded as a generalization of Lambert’s law to the case of colored media. It has been derived in a somewhat different form by Chekalinskaia [25] from scattering theory. In terms of the reflection (*r*), forward scatter (*t*), and absorption (*a*) constants of a single scattering layer(*a+r* +*t*=1) used by Chekalinskaia, the Rozenberg constants can be written

$$\begin{array}{cc} \beta =\frac{a}{r+t}; & Q=1+\frac{t}{r+t}. \end{array}$$ (27)

Il’ina and Rozenberg [26] have demonstrated the validity of eq (25) in several instances. Obviously in a highly absorbing medium where *β* ≫ 1, eq (25) further simplifies to

$$\frac{R_{0}}{R}\approx Q\beta .$$ (28)

In the other limit; i.e., where *β* ≪ 1, Rozenberg [15, 27] has shown that the reflectance can be written as an exponential of the form,

$$R_{\infty }(\mu ,\mu_{0})=\frac{I_{0}\mu_{0}}{\pi }h(\mu ,\mu_{0})\exp[-4\sqrt{q\beta }S(\mu ,\mu_{0})]$$ (29)

where *h*(*µ, µ*_0_), S(*µ, µ*_0_), and *q* are quantities which depend on the form of the scattering indicatrix. Romanova [28] has determined these quantities by exact solution of the radiative transfer equation.

### D. Exact Solutions

In problems of spectroscopy we often assume isotropic scatter. We know that in no case is single scatter actually isotropic [3], although the random distribution of anisotropic particles and scatter apparently tends toward an isotropic result [15]. Problems in highly anisotropic scattering media have been considered by some workers [29–34]. A detailed discussion of these solutions, often obtained by numerical computer methods, will not be discussed here.

For the simple case of isotropic scatter, the phase function (see eq (2)) can be written

$$p(\theta ,\phi ;\theta^{\prime }\phi^{\prime })=\omega_{0}=\frac{\sigma }{\sigma +\alpha }.$$ (30)

Here *ω*_0_ is known as the albedo of single scatter. It represents the fraction of the radiation lost by scattering in a medium where both absorption (*α*) and scattering (*σ*) take place. With this assumption, the equation of radiative transfer for a plane-parallel semi-infinite medium becomes independent of the azimuthal angle *ϕ* and we have

$$\mu \frac{dI(\tau ,\mu )}{d\tau }=I(\tau ,\mu )-\frac{1}{2}\omega_{0}\int_{-1}^{+1}I(\tau ,\mu^{\prime })d\mu^{\prime }.$$ (31)

The integral occurring in eq (31) may be approximated by a Gaussian quadrature, in which case a set of coupled linear differential equations is obtained,

$$\mu_{i}\frac{dI_{i}}{d\tau }=I_{i}=\frac{1}{2}\omega_{0}\sum_{j=-n}^{+n}a_{j}I_{j}.$$ (32)

The constants *a_j_* are Gaussian weighting functions given by

$$a_{j}=\frac{1}{P_{n}{\;}^{\prime }(\mu_{j})}\int_{-1}^{+1}\frac{P_{n}(\mu )}{\mu -\mu_{j}}d\mu$$ (33)

and *µ_j_* is one of the zeros of the Legendre polynomial, *P_n_*(*µ*).

Passing to the limit *n*→∞ gives an exact solution which Chandrasekhar [35] has shown to be of the form,

$$I_{r}(\mu )=\frac{\omega_{0}}{4}\Phi_{e}(\mu_{0})\frac{\mu_{0}}{\mu +\mu_{0}}H(\mu )H(\mu_{0}).$$ (34)

Here *I_r_*(*µ*) is the reflected intensity in the direction *µ* from a collimated incident beam in the direction *µ*_0_, whose flux per unit area normal to the beam is πΦ_e_(*µ*_0_). The *H*–integrals are defined by

$$H(\mu )=1+\frac{\mu H(\mu )}{2}\int_{0}^{1}\frac{H(\mu^{\prime })}{\mu +\mu^{\prime }}d\mu^{\prime }$$ (35)

and tables of them have been given by Chandrasekhar [35].

Giovanelli [36] has given explicit expressions for several cases of interest. The total reflectance for liedit incident in the direction *μ*_0_ is

$$R(\mu_{0})=1-H(\mu_{0}){\left(1-\omega_{0}\right)}^{1/2}$$ (36)

while that for diffused incident radiation is

$$R_{D}=1-2{\left(1-\omega_{0}\right)}^{1/2}\int_{0}^{1}\mu H(\mu )d\mu .$$ (37)

Tables of the first moment of the ***H*** – integral, which occurs in eq (37), have also been given by Chandrasekhar [35].

Exact solutions to the equation of radiative transfer can be derived for other phase functions as well. In general, the phase function may be expanded as a series of Legendre polynomials

$$p(\cos\;\theta )=\sum_{l=0}^{\infty }\omega_{l}P_{l}(\cos\;\theta )$$

where axial symmetry is assumed. Terms higher than first degree contribute very little [36, 37], and thus the approximate phase function.

$$p(\cos\;\theta )=\omega_{0}(1+x\cos\;\theta )(0\leq x\leq 1)$$

is sometimes used. Exact solutions are available for scatter according to this phase function also [36].

Equations (36) and (37) for isotropic scatter can be readily applied using tables given by Giovanelli [36], and they of course are of considerable theoretical interest since they represent exact solutions to which the various approximate theories can be compared. We do not expect all media to scatter isotropically, but we might expect the range of applicability of the equations to be extended if an appropriate average scattering coefficient were used. If we consider a diffuser in which each scattering center scatters light symmetrically about the direction of incidence, we may write [38]

$$\sigma_{\text{eff}}=(1-\bar{\mu })\sigma$$ (38)

where

$$\bar{\mu }=\frac{\int_{-1}^{+1}I(\mu )\mu d\mu }{\int_{-1}^{+1}I(\mu )d\mu }.$$ (39)

In this approach it is assumed that the same isotropic solutions (eqs (36) and (37)) may be used for arbitrary angular distributions of scatter, so long as the scattering is averaged according to eqs (38) and (39). Blevin and Brown [38] have shown that the reflectance curves are essentially the same for isotropic scatter or for scatter according to the phase functions 1 + *P*_1_(*μ*), 1 + *P*_2_(*μ*), and 1 + *P*_3_(*μ*). This suggests that the reflectance is not a sensitive function of the scattering indicatrix, and the isotropic solutions are in fact a good approximation for real scattering media.

## III. Statistical Theory

Continuum models, as we have seen, are somewhat limited. They involve the use of phenomenological constants with no obvious relationship in general to the fundamental constants with which we are familiar (molar absorptivity, refractive index, particle size and shape, etc.). Statistical theories, on the other hand, involve the construction of an appropriate model and the success of the theory depends on just how closely the model approximates real sample conditions.

It appears certain that one of the most severe limitations of continuum models is the assumption that they remain valid even when infinitesimal thicknesses are considered. This is in fact contrary to the assumption of homogeneous layers previously invoked (see section II–A), and it is this contradiction which is largely responsible for limiting the range of applicability of continuum models, as our subsequent discussion will show.

Let us return to eqs (8) and (9) and assume that we are now dealing with thin layers whose thickness is that of the individual particles. If we take layer *i* to be the first layer and layer *j* to be the combination of all the other layers of an *n*-layer sample, we have

$$t_{1,2,3,\ldots n}=\frac{t_{1}t_{2,3,4,\ldots n}}{1-r_{1}r_{2,3,4,\ldots n}}$$ (40)

and

$$r_{1,2,3,\ldots n}=r_{1}+\frac{t_{1}{\;}^{2}r_{2,3,4,\ldots n}}{1-r_{1}r_{2,3,4,\ldots n}}$$ (41)

Passing to the limit *n*→∞, we write

$$\begin{array}{l} t_{1,2,3,\ldots n}=t_{2,3,4,\ldots n}=T_{\infty }=0\\ r_{1,2,3,\ldots n}=r_{2,3,4,\ldots n}=R_{\infty } \end{array}$$

Equation (41) then becomes

$$R_{\infty }=r+\frac{t^{2}R_{\infty }}{1-rR_{\infty }}$$ (42)

where we have assumed that all layers are the same so the subscripts on *r* and *t* can be dropped. Equation (42) can be solved for *R*_∞_ to give an expression for the reflectance of an infinitely thick sample in terms of the reflectance and transmittance of a single layer. The result is

$$R_{\infty }=\frac{1+r^{2}-t^{2}}{2r}-\sqrt{{\left(\frac{1+r^{2}-t^{2}}{2r}\right)}^{2}-1}.$$ (43)

This equation is fundamental to essentially all statistical theories, the only difference being in the method used to calculate *r* and *t.*

We have seen that the Kubelka-Munk theory leads to a solution of the form,

$$\frac{{\left(1-R_{\infty }\right)}^{2}}{2R_{\infty }}=\frac{K}{S}$$

When this is solved for *R_∞_* we get

$$R_{\infty }=\frac{K+S-\sqrt{{\left(K+S\right)}^{2}-S^{2}}}{S}$$ (44)

which is not of the same form as eq (43).

We assume with Simmons [39] that the plane-parallel layers of the Kubelka-Munk model cannot be made infinitesimally small, but are restricted to layers of finite thickness *l*, where *l* may be interpreted as the mean particle diameter of the sample. Then the fundamental differential equations of the Kubelka-Munk theory (eqs (16) and (17)) are replaced by the finite difference equations:

$$\frac{dI_{+}}{dx}≅\frac{{\left(I_{+}\right)}_{i+1}-{\left(I_{+}\right)}_{i}}{l}=-\left(K+S\right){\left(I_{+}\right)}_{i}+S{\left(I_{-}\right)}_{i+1}$$ (45)

$$\frac{dI_{-}}{dx}≅\frac{{\left(I_{-}\right)}_{i+1}-{\left(I_{-}\right)}_{i}}{l}=\left(K+S\right){\left(I_{-}\right)}_{i+1}-S{\left(I_{+}\right)}_{i}$$ (46)

where the subscripts *i* and (*i*+1) refer to the *i*th. and (*i*+1)st sample layers, respectively. Now for an infinitely thick sample,

$$R_{\infty }=\frac{{\left(I_{-}\right)}_{i}}{{\left(I_{+}\right)}_{i}}=\frac{{\left(I_{-}\right)}_{i+1}}{{\left(I_{+}\right)}_{i+1}}$$

and Eqs (45) and (46) can be solved to give

$$R_{\infty }=\frac{2\left(S+K-KlS-K^{2}l/2\right)-\sqrt{4{\left(S+K-KlS-K^{2}l/2\right)}^{2}-4S^{2}}}{2S}$$ (47)

Equations (43) and (47) are identical if we make the following identifications:

$$\begin{array}{l} S=r/l\\ K=\left(1-r-t\right)/l=a/l \end{array}$$

where *a* is the fraction of the incident radiation which is absorbed by the layer.

The difference between the traditional and modified Kubelka-Munk solutions may be seen by writing eq (47) in the form,

$$F\left(R_{\infty }\right)=\frac{K}{S}-K-\frac{K^{2}l}{2S}=a\left(\frac{1}{r}-1\right)-\frac{a^{2}}{2r}.$$ (48)

It will be recognized that the difference lies in the addition of the last two terms. It is well known that a plot of *F*(*R*_∞_) versus *K* deviates from linearity for high values of *K* [7–9], and it appears that eq (48) can be used to explain the deviations in part. It should be recognized that the deviations at high values of *K* are probably a result of anomalous dispersion effects also, but eq (48) does represent an improvement in the range of validity and shows the need to consider the particulate nature of scattering media in developing a more precise theory by which absolute absorptivites can be determined.

### A. The Bodó Model

**Figure 3:**
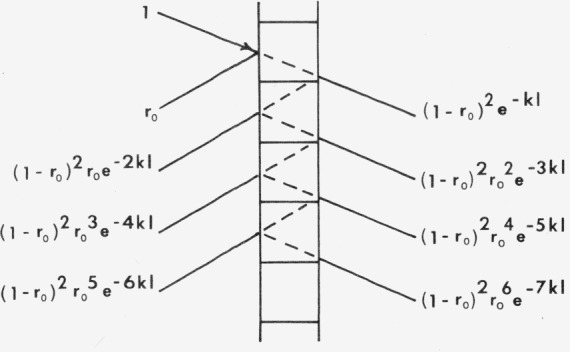
Reflectance and transmittance of a single layer of thickness l according to Bodó [40].

Bodó [40] used a procedure similar to that used to derive eq (43) for the derivation of *r* and *t.* We will denote the simple reflectance of the layer surface by *r*_0_, the absorptivity (defined through *I=I*_0_ exp (*−kx*)) by *k*, and the layer thickness (equivalent to the mean particle diameter) by *l* Then according to figure 3, the reflectance and transmittance of a single layer are given by

$$\begin{array}{l} r=r_{0}+{\left(1-r_{0}\right)}^{2}d^{-2kl}+{\left(1-r_{0}\right)}^{2}r_{0}{\;}^{3}e^{-4kl}+{\left(1-r_{0}\right)}^{2}r_{0}{\;}^{5}e^{-6kl}+\ldots \\ t={\left(1-r_{0}\right)}^{2}e^{-kl}+{\left(1-r_{0}\right)}^{2}r_{0}{\;}^{2}e^{-3kl}+{\left(1-r_{0}\right)}^{2}r_{0}{\;}^{4}e^{-5kl}+{\left(1-r_{0}\right)}^{2}r_{0}{\;}^{6}e^{-7kl}+\ldots \end{array}$$

Summing these series gives

$$r=\frac{r_{0}\left[1+\left(1-2r_{0}\right)\exp\left(-2kl\right)\right]}{1-r_{0}{\;}^{2}\;\exp\left(-2kl\right)}$$ (49)

$$t=\frac{{\left(1-r_{0}\right)}^{2}\exp\left(-kl\right)}{1-r_{0}{\;}^{2}\exp\left(-2kl\right)}.$$ (50)

Equations (49) and (50) together with eq (43) constitute the Bodó formulation, which is in fact equivalent to that of Stokes [1] and Girin and Stepanov [41]. Bodó [40] obtained good results with these formulae for powdered glass samples using the arbitrary assumption that *r*_0_=0.10. Karvaly [42] has shown that this was at least in part due to a particularly favorable position for the absorption band chosen for study, but in the general case, *R*_∞_ is a very sensitive function of *r*_0_.

Bauer [43] showed that in some cases the layers should be considered to have rough surfaces where total internal reflection can take place, and he has derived expressions analogous to eqs (49) and (50) for this case.

### B. The Johnson Model

**Figure 4:**
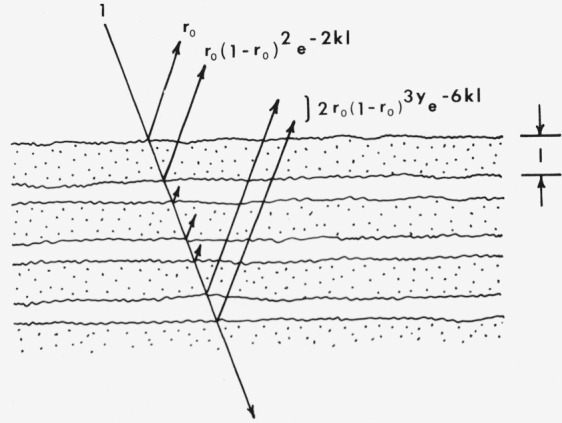
Model for Johnson analysis of reflectance from a scattering medium [44].

Johnson [44] has carried out the summation somewhat differently than Bodó, but with quite similar results (see fig 4). It is assumed that there are *p* layers and that the mean number of attenuating reflections which the rays undergo in the 2*p* traversals is *yp*, so that the reflectance is given by

$$R_{p}=r_{0}+2r_{0}\sum_{p}{\left(1-r_{0}\right)}^{yp}\exp\left(-2kpl\right).$$ (51)

Thus *y* can be regarded as an adjustable parameter which gives a semi-empirical account of multiple reflections as well as scattering losses. The sum for an infinite number of layers is

$$R_{\infty }=r_{0}+2r_{0}\frac{\exp\left[y\ln\left(1-r_{0}\right)-2kl\right]}{1-\exp\left[y\ln\left(1-r_{0}\right)-2kl\right]}$$ (52)

which is equivalent to eq (43) with

$$r=r_{0}\left[1+{\left(1-r_{0}\right)}^{v}\exp\left(-2kl\right)\right]$$ (53)

$$t={\left(1-r_{0}\right)}^{v/2}\exp\left(-kl\right).$$ (54)

The denominator of equation (52) can be expanded to give

$$R_{\infty }=r_{0}\left[\frac{2{\left(1-r_{0}\right)}^{v}\exp\left(-2kl\right)}{2kl-y\ln\left(1-r_{0}\right)}+1\right].$$ (55)

This result could also be obtained directly by integration of eq (51), which suggests that it may in fact be a more realistic representation of a real sample whose particles actually have a range of diameters.

It will be observed from figure 4 that *y* should be set equal to 4 for the case of no multiple reflections. In such a case only one half of the incident light is reflected, however. Johnson has suggested that *y* can be estimated from eq (55) by setting *R_∞_* = 1 for *k*=0. This gives

$$\frac{{\left(1-r_{0}\right)}^{y-1}}{y}=\frac{1}{2}+\frac{1}{4}r_{0}+\frac{1}{6}r_{0}{\;}^{2}+\frac{1}{8}r_{0}{\;}^{3}+\ldots .$$ (56)

from which *y*=2 is seen to be a satisfactory approximation for refractive indices smaller than 1.5. A smaller value of *y* is required for larger refractive indices. Companion and Winslow [43] have used a model similar to Johnson’s, but which includes all multiple reflections. The summation was carried out by computer and no explicit expression for the reflectance was given by these workers.

Johnson [44] also suggested that *r*_0_ be equated to 1.5 times the normal Fresnel reflectance. This is meant to account for the random distribution of particle surfaces and corresponds with an average incidence angle of approximately 30°. It was shown [44] that eq (55) yields absorption coefficients for KCl:Tl, KBr:Tl, and didymium glass which agree satisfactorily with those obtained by transmission measurements of the same materials.

### C. The Antonov-Romanovsky Model

**Figure 5:**
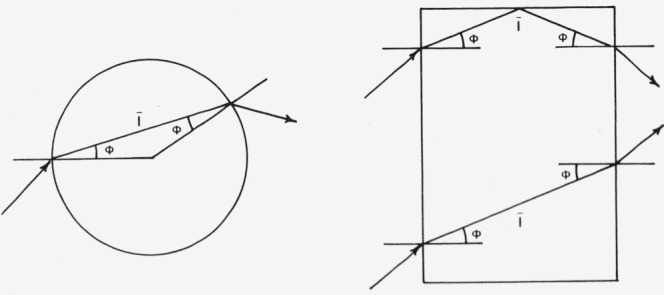
The Antonov-Romanovsky model for regular spheres and parallelepipeds [22].

Antonov-Romanovsky [22] has developed expressions which can be used to calculate the true absorption coefficient from reflectance measurements by connecting the Kubelka-Munk and Bodó theories. Antonov-Romanovsky treats two limiting cases of regularly-shaped sample particles, spheres and parallelepipeds (see fig. 5).

For spherical particles the radiation impinges on the surface from within at the same angle that it entered the particle, since the angles which the cord of a circle makes with respect to the surface normal must be the same. Therefore, total internal reflection is impossible, and $\bar{l}$ will approximate the particle diameter, *l*, actually being somewhat smaller.

In the case of the parallelepiped, some total internal reflection is possible, but most radiation probably exits through an opposite face without further reflection. In this case also it is obvious that $\bar{l}$ is approximately of the same magnitude as *l* but somewhat larger.

Thus for regularly shaped particles it is assumed that $\bar{l}\approx l$ and

$$k\approx \frac{1}{2l}\ln\frac{{\left(1-r_{0}\right)}^{2}-2r_{0}\left(1-2r_{0}\right)F\left(R_{\infty }\right)}{{\left(1-r_{0}\right)}^{2}-2r_{0}F\left(R_{\infty }\right)}.$$ (57)

This is an approximate form of the Bodó model which is valid for media in which *r*_0_^2^≪1.

For irregularly shaped particles it is assumed that the emerging radiation meets the surface of the particle with an equal probability for all angles. This will be the case if the mean number of reflections in the layer $\bar{m}$, is large:

$$\bar{m}\gg 1\;\left(n>1.5\right).$$

This condition requires that

$$kl_{f}\ll 1$$

where *l_f_* is defined as the “free” path length. The effective path length is then

$$\bar{s}=\bar{m}l_{f}$$ (58)

and it is assumed by Antonov-Romanovsky that

$$l_{f}\approx l/2.$$ (59)

The assumption that the emerging radiation is independent of angle allows us to divide it equally between the two sides of the layer whose thickness is *l* the mean particle diameter. We write

$$r-r_{0}=t$$ (60)

and from the law of conservation of energy,

$$\left(1-r_{0}\right)\left(1-\exp\left(-\bar{s}k\right)\right)=1-r-t.$$ (61)

Equations (60) and (61) can be solved to give

$$r=\frac{2r_{0}+\left(1-r_{0}\right)\exp\left(-\bar{m}kl/2\right)}{2}$$ (62)

$$t=\frac{\left(1-r_{0}\right)\exp\left(-\bar{m}kl/2\right)}{2}$$ (63)

Using these in eq (43) gives

$$k=\frac{2}{\bar{m}l}\ln\frac{{\left(1-r_{0}\right)}^{2}-\left(1-r_{0}\right)F\left(R_{\infty }\right)}{{\left(1-r_{0}\right)}^{2}-2r_{0}F\left(R_{\infty }\right)}.$$ (64)

### D. The Melamed Model

**Figure 6:**
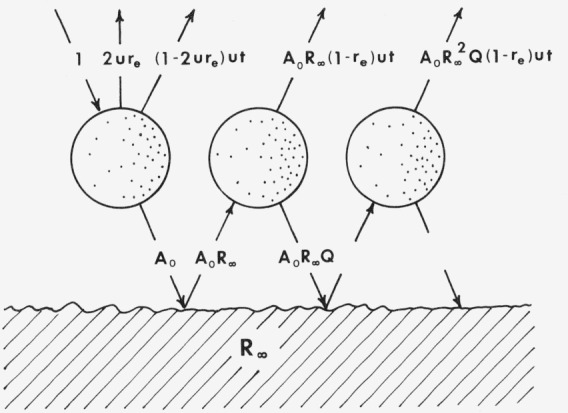
The Melamed Model for powder reflectance as viewed by Karvaly [47].

In contrast with the statistical theories previously discussed, Melamed carried out a summation over the reflectance and transmittance of individual particles rather than layers [46]. Some features of this model are better understood using an alternative derivation of Karvaly [47], which we follow here (see fig. 6). A single particle of the surface layer is shown in the figure; it is shifted laterally with each reflection to illustrate the path of the radiation.

**Figure 7:**
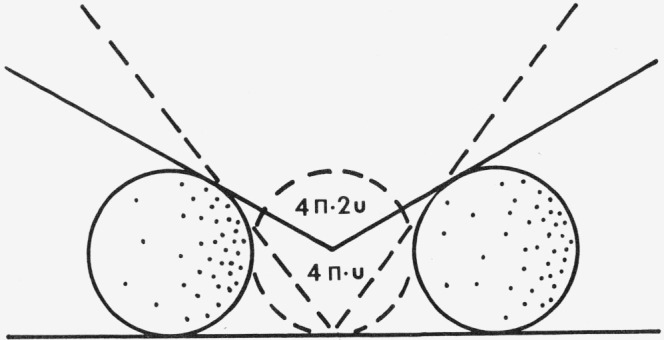
Illustration of the meaning of the radiant intensity factor u [47].

Of the diffuse radiation which is incident on a given surface particle, the fraction $2u{\bar{r}}_{e}$ is reflected, where the subscript *e* is used to indicate reflection of externally incident radiation. Here *u* is defined as the radiation emerging per unit solid angle from a particle. 4*πu* is the solid angle which would be observed from the second layer if a particle were removed (see fig. 7).

Of the radiant flux $\left(1-2u{\bar{r}}_{e}\right)$ which enters the particle, the part $\left(1-2u{\bar{r}}_{e}\right)ut$ contributes to the reflectance from the first internal reflection, and the part $A_{0}\equiv \left(1-2u{\bar{r}}_{e}\right)\left(1-u\right)t$ strikes the underlying layers, which are considered to form an infinitely thick powder mass of reflectance *R_∞_.* The transmission of an individual particle is represented by the symbol *t.* The part *A*_0_*R*_∞_ is reflected back into the layer, where $A_{0}R_{\infty }\left(1-{\bar{r}}_{e}\right)$ enters the particle and $A_{0}R_{\infty }{\bar{r}}_{e}$ is reflected back into the underlying layers. Of that part which entered the particle, $A_{0}R_{\infty }\left(1-{\bar{r}}_{e}\right)ut$ contributes to the reflectance and $A_{0}R_{\infty }\left(1-{\bar{r}}_{e}\right)\left(1-u\right)t$ is reflected downward again. This combines with that externally reflected to give $A_{0}R_{\infty }{\bar{r}}_{e}+A_{0}R_{\infty }\left(1-{\bar{r}}_{e}\right)\left(1-u\right)t=A_{0}R_{\infty }\left[{\bar{r}}_{e}+\left(1-u\right)t\right]\equiv A_{0}R_{\infty }Q$ as the flux reflected downward of which *A*_0_*R*_∞_^2^*Q* returns to the layer, etc.

Proceeding in this manner we can write the following expressions for the reflectance and transmittance of a layer for radiation from outside (*r*_1_ *t*_1_) and inside (*r_I_*, *t_I_*) the sample:

$$r_{1}=2u{\bar{r}}_{e}+\left(1-2u{\bar{r}}_{e}\right)u\cdot t$$ (65)

$$r_{I}={\bar{r}}_{e}+\left(1-{\bar{r}}_{e}\right)\left(1-u\right)t$$ (66)

$$t_{1}=\left(1-2u{\bar{r}}_{e}\right)\left(1-u\right)t$$ (67)

$$t_{I}=\left(1-{\bar{r}}_{e}\right)u\cdot t$$ (68)

Putting these expressions into (cf. equation (7))

$$R_{\infty }=r_{1}+\frac{t_{1}t_{I}R_{\infty }}{1-r_{I}R_{\infty }}$$

for an infinite number of layers gives

$$R_{\infty }=2u{\bar{r}}_{e}+\frac{\left(1-2u{\bar{r}}_{e}\right)\left(1-R_{\infty }{\bar{r}}_{e}\right)u\cdot t}{\left(1-R_{\infty }{\bar{r}}_{e}\right)-\left(1-u\right)\left(1-{\bar{r}}_{e}\right)R_{\infty }t}$$ (69)

The application of eq (69) requires some assumptions. Melamed assumes the sample to be composed of randomly-shaped particles which can be approximated as spheres. Then if we assume the absorbance is small (*kl≤*1) and that the radiation leaves the sphere isotropically,

$$u=\frac{u_{0}}{1-\left(1-2u_{0}\right)t}$$ (70)

where the shading factor *u*_0_ for close-packed spherical particles is 0.284. The transmittance of a single spherical particle including multiple internal reflections is written in terms of the internal reflectivity ${\bar{r}}_{i}$:

$$t=\frac{\left(1-{\bar{r}}_{i}\right)M}{1-{\bar{r}}_{i}M}$$ (71)

where

$$M=\frac{2}{{\left(kl\right)}^{2}}\left[1-\left(kl+1\right)\exp\left(-kl\right)\right]$$ (72)

is the transmittance of the particle for a single pass [48]. The mean external and internal reflectivities, ${\bar{r}}_{e}$ and ${\bar{r}}_{i}$, were calculated by Melamed as the averages over the Fresnel reflectivity *r*(*θ*),

$${\bar{r}}_{e}=2\int_{0}^{\pi /2}r(\theta )\sin\;\theta \;\cos\;\theta d\theta$$ (73)

$${\bar{r}}_{i}=\left(1-\sin^{2}\;\theta_{c}\right)+2\int_{0}^{\theta_{c}}r(\theta )\sin\;\theta \;\cos\;\theta d\theta$$ (74)

for an ideal diffusing surface obeying the Lambert cosine law [49]. Here *θ_c_* is the critical angle.

### E. The Fassler-Stodolski Model

Fassler and Stodolski [50–52] have pointed out that the Melamed theory is not only cumbersome to apply, but contains an inconsistency as well. It is assumed in the Melamed treatment that the radiation is distributed isotropically. However, we have seen in the previous section that when the Melamed equation is expressed in terms of the general layer model, we require different reflectances and transmittances for external and internal radiation (see eqs (65–68)).

A model which preserves some of the features of the Melamed theory, but which removes the inconsistency, has been constructed by Fassler and Stodolski as follows: we assume radiation to be externally incident on a particle layer. The part ${\bar{r}}_{e}$ is reflected at the surface, and the part $\left(1-{\bar{r}}_{e}\right)$ penetrates the layer. If we use the symbol *a* to represent the part which is absorbed in the layer, including the effect of multiple reflections, then the part (1−*a*) $\left(1-{\bar{r}}_{e}\right)$ leaves the particle either above or below the layer. We use *f_r_* and *f_t_* to represent the fractions of the radiation directed upward and downward, where

$$f_{r}+f_{t}=1.$$

Then the reflectance and transmittance of the layer are given by

$$r={\bar{r}}_{e}+f_{r}\left(1-a\right)\left(1-{\bar{r}}_{e}\right)$$ (75)

$$t=f_{t}\left(1-a\right)\left(1-{\bar{r}}_{e}\right)$$ (76)

For weakly absorbing systems it is assumed that *f_r_*=*f_t_*, corresponding with the Antonov-Romanovsky treatment of irregular particles. In the general case, *f_r_<f_t_.* Equations (75) and (76) with *f_r_=f_t_*=½ can be put into eq (42) to get

$$F\left(R_{\infty }\right)={\left(1-{\bar{r}}_{e}\right)}^{2}\frac{a}{1+{\bar{r}}_{e}-\left(1-{\bar{r}}_{e}\right)a}$$ (77)

*F*(*R*)_∞_ is once again the Kubelka-Munk function. Solving eq (77) for *a* gives

$$a=\frac{\left(1+{\bar{r}}_{e}\right)}{\left(1-{\bar{r}}_{e}\right)}\frac{F\left(R_{\infty }\right)}{\left[\left(1-{\bar{r}}_{e}\right)+F\left(R_{\infty }\right)\right]}.$$ (78)

If we assume the radiation within the powder layer to be isotropic, we can write (1−*t*) as the fraction absorbed, where *t* represents the transmittance of a given particle taking multiple reflections into account. Letting *u_s_* represent the fraction of the radiation from a given particle which remains within the layer, we have *u_s_t* as the part which enters a neighboring particle where (1−*t*)*u_s_t* is absorbed. Continuing in this way we once again generate a geometric series which can be summed to give the absorbance of the layer:

$$a=\frac{1-t}{1-u_{s}t}.$$ (79)

Following Melamed [46] the radiation is distributed among the fractions going up (*u_u_*), going down (*u_d_*), and going sideways (*u_s_*):

$$u_{u}+u_{d}+u_{s}=1.$$

Since we assume an isotropic distribution of the radiation, we can write *u_d_=u_u_=u*_0_, and so

$$u_{s}=1-2u_{0}$$

where once again *u*_0_=0.284 for spherical particles. Using this in eq (79) gives

$$a=\frac{\left(\frac{1}{t}-1\right)}{\left(\frac{1}{t}-1+2u_{0}\right)}.$$

Introducing eq (71) we can express this result in terms of the transmittance for a single pass,

$$a=\frac{\frac{1}{M}-1}{\frac{1}{M}-1+2u_{0}\left(1-{\bar{r}}_{i}\right)}.$$ (80)

The Duyckaerts equation for *M* (eq (72)) leads to a very complex expression, but with the Felder approximation [53],

$$M\approx \exp\left[-\frac{2}{3}kl\right],$$ (81)

equations (78) and (80) can be combined to give

$$kl=\frac{3}{2}\ln\frac{1+\left(\gamma -\delta \right)F\left(R_{\infty }\right)}{1-\delta F\left(R_{\infty }\right)}$$ (82)

where

$$\gamma =\frac{2u_{0}\left(1-{\bar{r}}_{i}\right)\left(1-{\bar{r}}_{e}\right)}{{\left(1-{\bar{r}}_{e}\right)}^{2}}$$

and

$$\delta =\frac{2{\bar{r}}_{e}}{{\left(1-{\bar{r}}_{e}\right)}^{2}}$$

The parameters *γ* and *δ* may be treated as constants which depend only on the refractive index, *n.* When *kl* is small, (*γ−δ*)*F*(*R_∞_*)*≪*1 and *δF*(*R_∞_*)*≪*1, and eq (82) simplifies to

$$kl=\frac{3}{2}\gamma F\left(R_{\infty }\right).$$ (83)

Thus there is a simple linear relationship between the true bulk absorption coefficient and the Kubelka-Munk function for weakly absorbing materials.

### F. The Simmons Model

Simmons [54] has used a simplified particle model to relate diffuse reflectance to fundamental optical constants without the use of the cumbersome equations which result from the more refined Melamed theory [46].

The law of conservation of energy requires that the total radiant flux impinging on a particle (Φ*_e_*) must equal that returning from it (Φ*_e_*′) except for the part which is absorbed:

$$\Phi_{e}=\Phi_{e}\prime +\Phi_{e}a.$$

If the spherical particles are assumed to scatter isotropically then Φ′*_e_=πl*^2^*M_e_* where *M_e_* is the radiant flux density, and

$$\Phi_{e}=\pi l^{2}M_{e}/\left(1-a\right).$$ (84)

Since the reflectance of an infinitely thick sample is independent of depth, we can write the two alternative expressions for the reflectance,

$$R=\frac{\left(\pi l^{2}M_{e}/2\right)}{\left(\pi l^{2}/2\right)\int_{0}^{\pi /2}M_{e}\left(\theta \right)\sin\;\theta d\theta }$$ (85)

$$R=\frac{\left(\pi l^{2}/2\right)\int_{\pi /2}^{\pi }M_{e}\left(\theta \right)\sin\;\theta d\theta }{\left(\pi l^{2}M_{e}/2\right)}$$ (86)

Φ*_e_* is the total flux integrated over the whole sphere, so eq (85) and (86) combine to give

$$\Phi_{e}=\left(\pi l^{2}/2\right)\int_{0}^{\pi }M_{e}\left(\theta \right)\sin\;\theta d\theta =\pi l^{2}M_{e}\left(R+R^{-1}\right)/2.$$

Equating this to eq (84) gives

$$R=\left[1-{\left(2a-a^{2}\right)}^{1/2}\right]/\left(1-a\right).$$ (87)

We let *M* equal the absorption from a single pass through the spherical particle. Of the incident radiation the fraction $\left(1-{\bar{r}}_{e}\right)$ penetrates the particle where the fraction $\left(1-{\bar{r}}_{e}\right)$ (1−*M*) is transmitted, and of this, the fraction ${\bar{r}}_{i}$ is internally reflected. The total fraction absorbed following an infinite number of such inter-reflections is

$$\begin{array}{l} a=\left(1-{\bar{r}}_{e}\right)\left[\left(1-M\right)+{\bar{r}}_{i}M\left(1-M\right)+{\bar{r}}_{i}^{2}M^{2}\left(1-M\right)+\ldots \right].\\ =\frac{\left(1-{\bar{r}}_{e}\right)\left(1-M\right)}{1-{\bar{r}}_{i}M} \end{array}$$

Again using the Felder approximation [53] (equation (81)) for spherical particles gives

$$a=\frac{\left(1-{\bar{r}}_{e}\right)\left[1-\exp\left(-2kl/3\right)\right]}{1-{\bar{r}}_{i}\exp\left(-2kl/3\right)}.$$ (88)

A simple expression which is valid for small values of *a* can be derived by noting that eq (87) represents the leading terms of a series expansion of the form,

$$R=1-{\left(2a\right)}^{1/2}+a-\ldots =\exp\left[-{\left(2a\right)}^{1/2}\right].$$ (89)

If the exponential terms in Equation (88) are likewise expanded we find

$$a\approx \frac{\left(1-{\bar{r}}_{e}\right)2kl/3}{1-{\bar{r}}_{i}+2kl{\bar{r}}_{i}/3}\approx \frac{1-{\bar{r}}_{e}}{1-{\bar{r}}_{i}}\frac{2kl}{3}.$$ (90)

It can be shown that [54]

$$\frac{1-{\bar{r}}_{e}}{1-{\bar{r}}_{i}}=n^{2}.$$

With this result eq (90) can be combined with eq (89) to give

$$R=\exp\left[-2n{\left(kl/3\right)}^{1/2}\right].$$ (91)

This gives a simple relationship between reflectance and the fundamental optical parameters which has been shown to be valid for weakly absorbing samples [54].

It was found that the above simple particle theory disagrees with the Melamed theory for large refractive indices [55]. The discrepancy has been attributed to a breakdown of the assumption that the externally incident radiation reflected at the particle surface is scattered equally in the upward and downward directions, an assumption which obviously becomes more questionable as *n* increases. By correcting the simplified particle model for this non-isotropic reflection of incident radiation, the relationship

$$R=\frac{1+{\bar{r}}_{e}\left(1-a\right)/2-\sqrt{\left(2a-a^{2}\right)\left(1-{\bar{r}}_{e}{\;}^{2}/4\right)}}{{\bar{r}}_{e}/2+1-a}$$ (92)

was derived, which gives reflectances which agree well with those of the Melamed theory for large refractive indices [55].

Johnson [56] has pointed out that the Melamed theory [46] predicts that the diffuse reflectance decreases with increasing values of the relative refractive index, *n.* The same applies to the simplified particle model described above. Such behavior is reasonable for large values of *n* but for values near unity it has been shown [56] experimentally that *R*→0 as *n*→1, as would be expected.

The failure of the Melamed theory and the simplified particle model theory of Simmons for *n* values near unity is thought to be due to the invalid assumption that radiation is returned equally in all directions from a given particle [57]. It is found that for a rough-surfaced particle; i.e., one whose surface obeys the Lambert cosine law, three-fourths of the incident radiation reverses its direction while one-fourth does not [55]. It is assumed that the radiation which is incident at a particle surface may be divided into that which is randomly scattered and that which is transmitted. If that part which is scattered becomes isotropically distributed and the direction of the transmitted radiation is essentially unchanged, then the following expressions can be derived:

$$r=\frac{3}{4}{\bar{r}}_{e}+\left(1-{\bar{r}}_{i}\right)\left(1-{\bar{r}}_{e}\right)\left(\frac{1}{2}t\right)\left[\frac{1}{1-{\bar{r}}_{i}t}+\frac{\left(1-\frac{1}{2}{\bar{r}}_{i}\right)\left(1-\frac{1}{2}{\bar{r}}_{e}\right)}{1+\frac{1}{2}{\bar{r}}_{i}t}\right]$$ (93)

$$t=\frac{1}{4}{\bar{r}}_{e}+\left(1-{\bar{r}}_{i}\right)\left(1-{\bar{r}}_{e}\right)\left(\frac{1}{2}t\right)\left[\frac{1}{1-{\bar{r}}_{i}t}+\frac{\left(1-\frac{1}{2}{\bar{r}}_{i}\right)\left(1-\frac{1}{2}{\bar{r}}_{e}\right)}{1+\frac{1}{2}{\bar{r}}_{i}t}\right].$$ (94)

These equations for *r* and *t* are used with eq (43) to calculate the reflectance. It is found that the agreement with Johnson’s data [56] is only qualitative, although the solution does have the feature that *R* → 0 as *n* → 1.

## IV. Discussion of the Various Theories

If we assume isotropic radiation and the simplest Gaussian quadrature *n*=1 in eq (32), we obtain the Kubelka-Munk equations (16) and (17), with

$$S=\frac{\sigma }{\sigma +\alpha }\text{and}\;K=\frac{2\alpha }{\sigma +\alpha }.$$

Thus the Kubelka-Munk function may be regarded as a first approximation to the complete solution for the equation of radiative transfer. Detailed comparisons show that the two solutions differ by no more than a few percent, and even this difference is of little consequence in a comparative-type measurement [12].

The Kubelka-Munk theory is in fact quite general, and encompasses many other two-constant theories which have been derived to suit certain select experimental conditions [7,23]. With the Kubelka-Munk solution expressed in terms of hyperbolic functions, it is possible to write formulas for many specific applications, and to show the connection with several other theories. It was shown explicitly by Kubelka [21] that the various equations of Gurevič [20] and Judd [58] could be derived from the Kubelka-Munk equations. The Gurevič and Judd theories are less general, however, and do not encompass completely the Kubelka-Munk results.

The Gurevič layer model is sufficiently general to allow us to use it in making some general observations concerning the continuum models and their range of applicability. Since Kubelka [21] has derived eq (12) of Gurevič in terms of the hyperbolic solution, we have a connection between the parameters used in the two cases. In particular,

$$L\equiv {\left(a^{2}-1\right)}^{1/2}S=bS$$

where *a* is defined by eq (18), and *R*_∞_ has the same meaning in the two theories. *L* and *R*_∞_ are related in turn to the Gurevič constants *K*_1_ and *K*_2_ through eqs (14) and (15).

It will be recalled that *K*_1_ and *K*_2_ are positive definite constants which are characteristic of the light scattering medium (see eqs (10) and (11)). That is, we assumed thin homogeneous layers where *T* and *R* depend linearly on *x.*

We find, in fact, that eqs (10) and (11), and correspondingly all continuum model results, are applicable only within a limited range. From eq (11) we have the obvious restriction that *K*_2_*dx<*1. It is equally obvious that the equations do not apply when *dx* is made arbitrarily small, since the assumption of homogeneous layers requires it to be large compared with the dimension *l* of the scattering particles [14, 39]. Thus we have [16]

$$l\ll dx<\frac{1}{K_{2}}$$

which can alternatively be written

$$K_{2}l\ll 1.$$

We see that the attenuation due to absorption within a layer of thickness *l* must be very small compared with unity, and we are restricted to weakly absorbing and weakly scattering materials. It can in fact be shown by expanding eqs (12) and (13) in series as a function of *x* that eqs (10) and (11) follow only if one assumes that the medium scatters and absorbs weakly [59].

Several authors have investigated the relationship between continuum and statistical theories, ter Vrugt [60] compared the Kubelka-Munk and Bodó theories and found that the absorption constants determined by the two methods agree quite well. It was shown that for weak absorbers the parameters are related by

Kx=kl (95)

and

$$Sx=\frac{2r}{1-r}$$ (96)

If the crystals are not too irregular, the mean path length of radiation in the particle will be approximately equal to the particle diameter and the absorption constants described by the two methods will be nearly equal. A more general relationship is

$$\exp\left(2kl\right)=\frac{2\left(1-r\right)}{F\left(R_{\infty }\right)/F\left(r\right)-1}$$ (97)

which is valid for materials of medium absorption as well.

Poole [61] has shown that the Kubelka-Munk and Melamed theories lead to the same results for small values of the refractive index, *n.* In general, the absorption constants determined by the two methods are not the same and a curve is given by Poole which relates them as a function of *n.* It was subsequently shown by Karvaly [47] that the proportionality factor can be expressed analytically as

$$\frac{2}{3}n^{2}\frac{1-r_{e}}{1+r_{e}}$$ (98)

and that this relationship can in fact be used to estimate *n* for a powdered material.

Several of the theories were compared by Companion [62] in an attempt to interpret reflectance spectra of metal oxides such as NiO and V_2_O_5_. It was found that the Kubelka-Munk theory gives unexplained distortions and peak shifts, the Melamed theory requires a shading factor *u*_0_~0.1 rather than 0.284 which is appropriate for spherical particles, and the Johnson estimate of the factor *y* is not a good approximation. These discrepancies are all thought to be a result of the failure of the theories to explicitly include anomalous dispersion effects.

Karvaly [47] has shown that the Johnson model is a limiting case of the Bodó theory which should be valid in the limit of large particles or materials of large optical thickness. Antonov-Romanovsky’s formulae for regularly shaped particles are likewise derivable from the Bodó theory [47]. Careful measurements of the reflectance of two didymium glass powder samples ( $\bar{l}=7.3$ and 12.2 *μ*m) by Karvaly and Pinter [63] have shown that the Bodó and Antonov-Romanovsky theories give absorption constants which are accurate to about ±35 percent, whereas the Johnson and Melamed theories did not lead to satisfactory results in this case.

As mentioned above, the Melamed theory fails to predict that *R*→0 as *n*→1, as must be the case since there are then no scattering centers in the medium [56]. In the Johnson and Bodó models the path of the radiation is altered by internal and external reflections and refractions at particle surfaces, whereas in the Melamed model it is diverted by “scattering” according to the Lambert cosine law. In all three cases it is assumed that *l≫λ.* This allows scattering to be neglected in the Johnson and Bodó models. The dependence of *R* on *n* in the Melamed theory is apparently due to the scattering of radiation internally incident on the particle surface which is taken to be independent of *n.* It is interesting to note that the three theories give about the same reflectance for refractive indices 1.7<*n*<2.0.

It is of course not necessary to assume that the particles are rough-surfaced, i.e., that they scatter according to the Lambert cosine law. In the other limit they may be approximated as smooth-surfaced spheres whose reflection and refraction is governed by Fresnel’s laws. This case has been treated by Simmons [64] and represents the opposite extreme between which most real systems lie.

The recently published modified particle model theory of Simmons [57] (Equations (93) and (94)) removes the discrepancy in the Melamed treatment. This modified particle model theory is thought by Simmons to be the most nearly correct of all diffuse reflectance theories [39]. It may very well be true, but its general acceptance must of course await a thorough testing under a variety of conditions by various workers.

The Fassler and Stodolski theory also shows considerable promise, but has not yet been thoroughly tested. It does lend itself to the investigation of important effects such as the influence of particle size distribution on reflectance properties [52, 65], and will probably receive considerable attention in the future.

## V. Interpretation of Reflectance Parameters

One of the difficulties with weighing the relative merits of the various reflectance theories is the necessity of comparing them over a wide range of experimental conditions. In general, the data are rather incomplete and we must content ourselves with pointing out the physical significance of the parameters used. We will do this in the present section, together with making some remarks concerning the characteristics of appropriate model systems.

The internal transmittance of a powder layer in terms of hyperbolic functions has been given by Kubelka, [21]

$$T_{i}=\frac{b}{\left(a\;\sinh\;bSX+b\;\cosh\;bSX\right)}$$

where *a* and *b* are previously defined (eqs (18) and (21)). If we assume that *S* is small compared with *K*,

$$a\approx b\approx \frac{K}{S}$$

and

$$T_{i}\approx \exp\left(-KX\right).$$

Under these conditions the transmittance follows a Lambert-type law and *K* can be regarded as an absorptivity characteristic of the substance.

The Kubelka-Munk constant *K* for a scattering medium is not equivalent to that which would be determined by transmission measurements on an identical material without scatter, however. For a beam traveling through the infinitesimal layer *dx* at an angle *θ*, the path length in the layer is *dx/*cosθ, and thus the mean path length of radiation traveling downward is

$$dx\int_{0}^{\pi /2}\frac{\partial I_{+}}{I_{+}\partial \theta }\frac{d\theta }{\cos\;\theta }$$ (99)

where *∂I_+_/∂θ* is the angular distribution of the intensity in the positive *x* direction. We of course have an analogous expression for the *I_−_* component. If we assume that the medium is an ideal diffuser the intensity is the same in all directions, and the angular distribution of intensity through a given plane becomes

$$\frac{\partial I_{+}}{\partial \theta }=I_{+}\sin2\theta$$

so that eq (99) becomes equal to 2*dx.* Thus the effective path length in the scattering layer is twice the normal layer width because of the random angular distribution, and the apparent absorptivity should be larger by a factor of two.

The above result has been tested using colored filter glass by Kortüm and Oelkrug [66]. By measuring both the reflectance and scattered transmission of thin layers, both the scattering and absorption coefficients can be determined. Even though the powder cannot be packed to the same density as the original glass, the ratio of reflectance to transmittance values exceeded the theoretical limit, being in the range *K*/*k~*2.6–2.9. This difference was attributed to effects of total internal reflection which should increase the path length even further [8]. There are numerous factors which can cause deviations from the factor of two in non-ideal samples, and these factors have been discussed by Van den Akker [67]. We will continue to restrict our discussion to ideal systems, however.

The statistical theories predict this ratio between absorptivity determined by reflectance and transmittance with varying degrees of success. As already mentioned, ter Vrugt [60] found them to be equal for weak absorbers (cf. eq (95)). The Antonov-Romanovsky theory [22] gives

$$\frac{K}{k}=\frac{1-r_{0}}{1+r_{0}}.$$

For the BG 24 filter glass used by Kortüm and Oelkrug [66] the ratio is 0.92, which does not agree well with the experimental values.

For low refractive indices Poole [61] showed that the Kubelka-Munk and Melamed theories agree with a proportionality factor of 2.7, but much higher values are required for higher *n* values (cf. eq (98)). Fassler and Stodolski [68] have also derived a relationship between *K* and *k* which involves the refractive index of the medium:

$$\frac{K}{k}=\frac{1}{3u_{0}}\frac{n^{2}}{\left(1-{\bar{r}}_{e}\right)}.$$

The ratio is calculated to be in the range 2.9–3.1 for the filter glass used by Kortüm and Oelkrug [66], which is in excellent agreement with the experimental values. The *n*^2^-dependence of *K/k* has also been calculated by Simmons [71] from his particle model theory. The result is.

$$\frac{K}{k}=\frac{2n^{2}}{3}$$

which also agrees fairly well with the Kortüm and Oelkrug data [68].

Multiple scattering in dense media is a phenomenon which is not completely understood. Mie [3] has given an exhaustive treatment of single scatter and has shown that the scattering varies from the *λ*^−4^- dependence of Rayleigh scatter for very small particles to a *λ*-independence for particles which are large compared with the wavelength of the radiation. There is no *a priori* reason to expect that these results would apply quantitatively in the case of multiple scatter, but it has in fact been found that there is a rather close correlation. Kortüm and Oelkrug [66] found that the scattering varies as ~*λ^−^*^3.5^ for *l*<*λ*, as ~λ^−1^ for *l*≈λ, and as ~λ^0^ for *l<λ* where *l* is the mean particle diameter. It is safe to assume that the scattering coefficient *S* is independent of wavelength in most studies, but deviations are sometimes observed in the short wavelength end of the spectrum which are probably due to a breakdown of this assumption [69].

The scattering coefficient has been found in several studies to be inversely proportional to the mean particle diameter *l*, [70–72] the proportionality factor depending apparently on the nature of the material being studied. Using the simplified particle model, Simmons was able to derive the result [69].

$$S=l^{-1}$$

This should not be taken as much more than an order of magnitude estimate, since a proportionality factor somewhat larger than unity is apparently more appropriate in many cases [66]. The modified particle model gives [39]

$$S=l^{-1}\left\{\frac{3{\bar{r}}_{e}}{4}+\frac{t}{2}\left(1-{\bar{r}}_{e}\right)\left(1-{\bar{r}}_{i}\right)\left[\frac{1}{1-{\bar{r}}_{i}t}-\frac{\left(1-{\bar{r}}_{i}/2\right)\left(1-{\bar{r}}_{e}/2\right)}{1+{\bar{r}}_{i}t/2}\right]\right\}.$$

Although this result appears to not yet be thoroughly tested, the term in braces is said to be relatively insensitive to changes in absorptivity [41], and probably varies significantly only with changes in refractive index, *n.* The same is true of the Antonov-Romanovsky result [22],

$$S=\left(\frac{2r_{0}}{1+r_{0}}\right)l^{-1}$$

and that derived by ter Vrugt [60] (see eq (96)). Quantitative calculations with the Antonov-Romanovsky expression give scattering constants that are too small by about a factor of three [47].

By taking the particle size distribution into account, Fassler and Stodolski [65] were able to calculate scattering coefficients which agree very well with those determined experimentally. Their expression is

$$S{\bar{l}}^{\prime }=\frac{\frac{1+\bar{r}*-\xi }{1-\bar{r}*-\xi }-a-\left(2f_{t}-1\right)\left(1-a\right)}{\frac{1+\bar{r}*+\xi }{1+\bar{r}*-\xi }+\left(2f_{t}-1\right)\left(1-a\right)}$$ (100)

where *a* and *f_t_* have their previously defined meanings, it being recognized that the factor (2*f_t_*-l) defines the anisotropy of radiation in the particle layer. *ξ* is the effective cross section for holes in the particle layer, $\bar{r}*$ is the mean external reflection coefficient for side scatter taking account of holes and back scatter, and $\bar{l}\prime$ is a parameter which is related to the mean particle diameter. For a particle size distribution function of the form

$$f\left(l\right)=Al^{B}e^{-Bl/\bar{l}}$$

$\bar{l}\prime$ is given by

$${\bar{l}}^{\prime }=\frac{B+3}{B}\bar{l}.$$

Fassler and Stodolski [65] emphasize the fact that the scattering is particle size-dependent, so that for quantitative reflectance spectroscopy it is essential to have a set of standards with known, small particle size distribution and of various mean particle diameters. Then it is possible to mix the sample with a standard of similar particle size so that the scattering characteristics are not greatly changed. This is important for comparative measurements.

The effect of particle shape is also important. It appears at the present time that there is no better way to test the various reflectance theories than to make transmission and reflectance measurements on a given glass sample before and after grinding, respectively. The characteristics of glass samples subjected to various grinding conditions have been studied by Karvaly and Pintér [73]. These factors will not be discussed here, but it should be emphasized that the proper definition of reflectance standards must certainly quantify not only size and shape, but the precise nature of any adsorbent materials and the surface conditions as well.

## Footnotes

1 Figures in brackets indicate the literature references at the end of this paper.

## List of Principal Symbols Used

(Note: Where a given letter is used in both capital and lower case form (e.g., *r,R* and *t,T*) the capital letter refers to the macroscopic observable and the lower case letter to the corresponding variable for an individual particle or layer of the material. A bar over a given symbol means the average value for that variable).

*a*: total absorption of a single particle (layer); also $\frac{S+K}{S}$ (equation (18))
*a_j_*: Gaussian weighting function (equation (33))
$\begin{array}{c} a_{it}\\ a_{ijt} \end{array}\;\}$: Rozenberg constants (equation (23) ff.)
*b*: (*a*^2^–1)^1/2^ (equation (21))
*C_i_*: *I_i_/I*_1_ polarization of radiant beam (equation (24))
*τ*: fraction of radiation reflected (equation (75))
*f_t_*: fraction of radiation transmitted (equation (76))
*h*(*µ,µ_0_*): Rozenberg constant (equation (29))
*H*(*µ*): *H*-integral of Chandrasekhar (equation (35))
*I*: radiant intensity
*I′*: component of source function for self-radiation (equation (5)) *j* scattering function (equation (2))
*J*: source function (equation (4))
*k*: absorptivity
*K*: Kubelka-Munk absorption constant (equations (16) and (17))
*K*_1_*K*_2_: Attenuation constants in Gurevič layer model (equations (10) and (11))
*l*: particle diameter or layer thickness
*l_f_*: “free” path length of Antonov-Romanov-sky (equation (58))
L: Gurevič constant $=\sqrt{K_{2}{\;}^{2}-K_{1}{\;}^{2}}$
$\bar{m}$: mean number of reflections
M: transmittance of a particle for a single pass
M*_e_*: radiant flux density
*p*: phase function (equation (2))
*P_n_*(*µ*): Legendre polynomial
*q*: Rozenberg constant (equation (29))
*Q*: Rozenberg multiple reflection constant (equation (26))
*r*: total reflectance of a single particle (layer)
*r*_0_: simple reflectance of a single particle (layer) surface
$\bar{r}*$: mean external reflection coefficient for side scatter
*r_e_*: reflectance of a particle (layer) for externally incident radiation
*r_i_*: reflectance of a particle (layer) for internally incident radiation
*R*: reflectance of a (macroscopic) layer
*R*_∞_: reflectance of an infinitely thick layer
$\bar{s}$: effective path length (equation (58))
*S*: Kubelka-Munk scattering constant (equations (16) and (17))
*S*(*µ,µ*_0_): Rozenberg weak scatter constant (equation (29))
*t*: total transmittance of a single particle (layer)
*T*: transmittance of a (macroscopic) layer
*u*: radiation emerging from a particle per unit solid angle
*u*_0_: shading factor (equation (70))
*u_d_*: fraction of radiation emerging from a particle in a downward direction
*u_s_*: fraction of radiation emerging from a particle in a sideways direction
*u_u_*: fraction of radiation emerging from a particle in an upward direction
*x*: length
*y*: Johnson multiple reflection factor (equation (51))
*α*: absorption coefficient
*β*: *α/σ* (equation (23))
*γ*: Fassler-Stodolski constant (equation (82))
*δ*: Fassler-Stodolski constant (equation (82))
*θ*: polar angle
*κ*: attenuation coefficient = *α*+ *σ* (equation (1))
*λ*: wavelength
*µ*: cos *θ*
*µ_j_*: one of the roots of the Legendre polynominal *P_n_*(*µ*)
*ξ*: effective hole cross section (equation (100))
*ρ*: density
*σ*: scattering coefficient (equation (2))
*τ*: optical thickness (equation (3))
*ϕ*: azimuthal angle radiant flux
*ω*_0_: albedo of single scatter (equation (30))
